# Case Report: Contezolid-containing regimens for disseminated Bacillus Calmette–Guérin disease in three children with primary immunodeficiency

**DOI:** 10.3389/fped.2026.1928342

**Published:** 2026-10-02

**Authors:** Zhen Zhang, Junchi Xu, Li Gao, Junjia Liu, Zhijian Ye, Yong Xu, Jianping Zhang, Xin Yu

**Affiliations:** 1 Department of Lung Disease, The Fifth People’s Hospital of Suzhou/The Affiliated Infections Diseases Hospital, Suzhou Medical College of Soochow University, Suzhou, China; 2 Department of Hematology, Children’s Hospital of Soochow University, Suzhou, China

**Keywords:** case report, children, contezolid, disseminated BCG disease, primary immunodeficiency

## Abstract

**Introduction:**

Disseminated Bacillus Calmette–Guérin (BCG) disease is a rare but life-threatening complication in children with primary immunodeficiency (PID). Linezolid (LZD) is effective but limited by myelosuppression. Contezolid (CZD), a novel oxazolidinone, has shown better hematological safety in adults, but pediatric data are scarce. This retrospective case series aims to provide real-world experience on CZD use in this challenging population.

**Case description:**

We report three children (aged 3–9.5 months) with PID (Wiskott–Aldrich syndrome, X-linked severe combined immunodeficiency, and IL-12 receptor β1 deficiency) who developed clinically presumed or confirmed disseminated BCG disease after routine vaccination. All received CZD-containing regimens (12–20 mg/kg/dose, q12 h) alongside other antimycobacterial agents and hematopoietic stem cell transplantation (HSCT).

**Outcomes:**

Over 11–12 months of CZD-containing multidrug therapy followed by 1 year of post-treatment telephone follow-up, all patients achieved satisfactory clinical resolution of infectious lesions and no clinical relapse was observed. No CZD-attributable hematological, hepatic, renal, or neurological adverse events occurred. However, because all patients received concurrent HSCT and multiple antimycobacterial agents, the independent contribution of CZD cannot be determined.

**Conclusion:**

CZD-containing regimens were well tolerated and associated with clinical improvement in these complex cases. CZD may serve as a valuable alternative when LZD is poorly tolerated. However, definitive attribution of efficacy to CZD alone is precluded by concurrent HSCT and multi-drug therapy.

## 1 Introduction

Among children with inborn errors of immunity (IEI), BCG vaccination can trigger disseminated disease, with a reported incidence of 0.15 per 100,000 doses in some Chinese regions (1). Therapeutic choices for disseminated BCG disease remain constrained. First-line agents including isoniazid (INH), rifampicin (RFP) and ethambutol (EMB) are often insufficient, and second-line drugs like LZD are restricted by dose-dependent myelosuppression, peripheral neuropathy, and lactic acidosis, especially in the post-HSCT aplastic phase (2). CZD is a novel oxazolidinone with potent *in vitro* activity against *Mycobacterium tuberculosis* complex (MTBC) and a superior hematological safety profile in adults, as demonstrated in a recent randomized trial (3). Multiple case series have confirmed that switching from LZD to CZD resolves or improves adverse events in approximately 90% of intolerant patients while maintaining clinical efficacy (4, 5). To date, reports on CZD for pediatric disseminated BCG disease remain scarce. A recent retrospective study described CZD use in pediatric HSCT recipients with PID and mycobacterial infections (6), and a single case abstract has been published (7).

The unique feature of our cases lies in the diversity of underlying genetic defects (*WAS*, *IL2RG*, *IL12RB1*) and the detailed retrospective longitudinal documentation of CZD tolerability in the context of HSCT and multi-drug regimens. This report adds to the emerging literature by providing extended follow-up data and a nuanced discussion of confounders. Importantly, this is a retrospective case series. Patients were not prospectively enrolled or followed under a research protocol.

## 2 Case description

This was a retrospective case series. The three patients were identified from the Children's Hospital of Soochow University between August 2023 and November 2025. Data were collected retrospectively from electronic medical records, laboratory results, imaging studies, medication records, and telephone follow-up. Diagnosis followed expert consensus (8), requiring BCG vaccination, confirmed PID, disseminated infection, and microbiological/molecular evidence of MTBC. All had metagenomic next-generation sequencing (mNGS) confirmation; in Case 3, species-level identification of *Mycobacterium bovis* BCG was obtained. Written informed consent was obtained from parents/guardians.

### 2.1 Case 1

A male infant aged 3 months 20 days was admitted with a 2-month history of intermittent fever. Recurrent fever had been present since 14 days of age. He had received routine BCG vaccination at birth. At approximately 1.5 months of age, an ulcerated mass was observed in the left axilla (Figure 1A). Genetic testing performed at an external facility identified a *WAS* gene mutation (NM_000377.3: c.809T >C, p.Leu270Pro), inherited from the mother, confirming a diagnosis of Wiskott–Aldrich syndrome (WAS). mNGS of the purulent discharge detected MTBC, consistent with BCG-related infection in the context of recent vaccination and underlying immunodeficiency. An anti-mycobacterial regimen of INH, RFP, and EMB was initiated, and the fever subsided with partial stabilization of the axillary mass.

**Figure 1:**
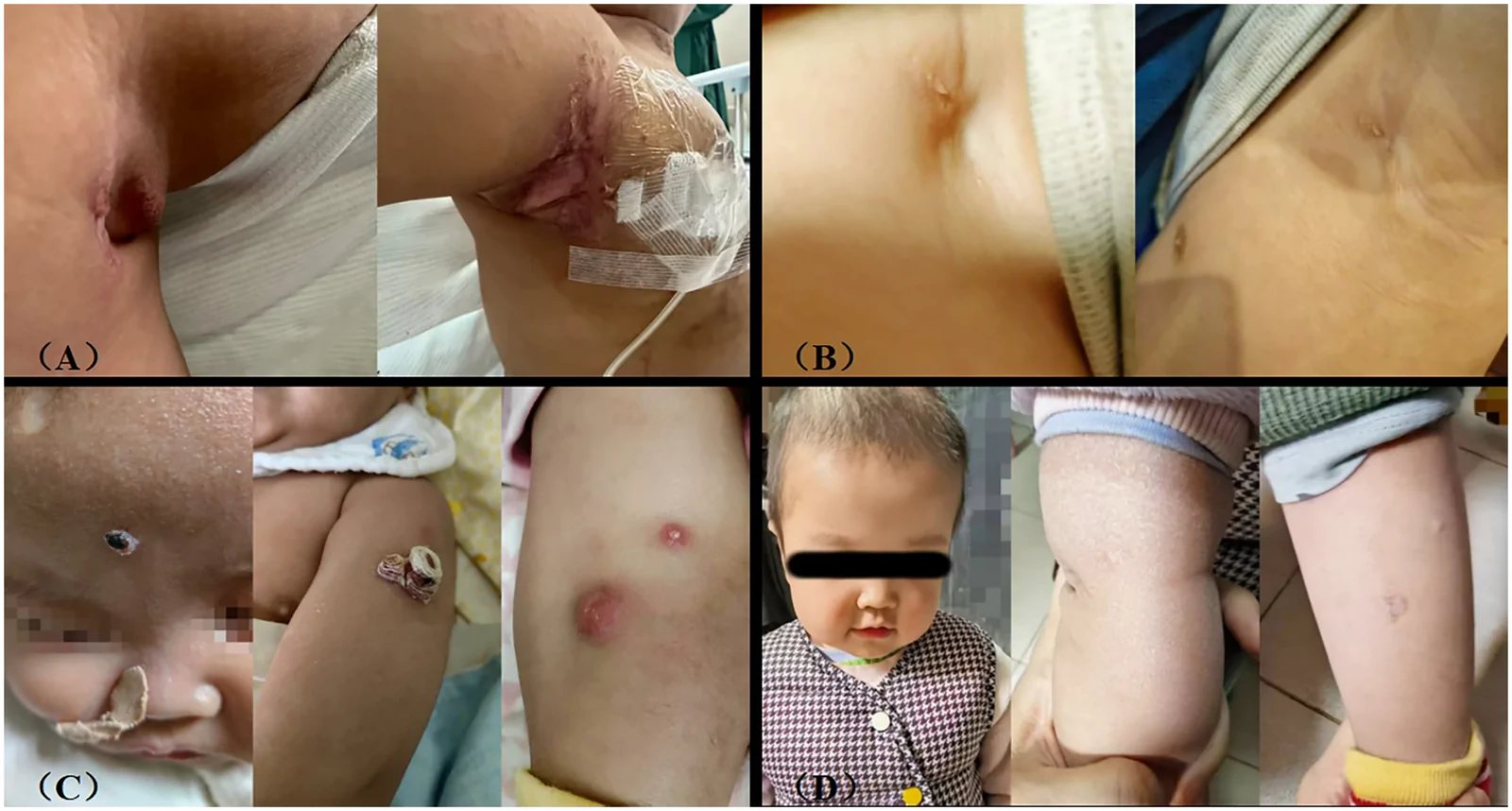
Clinical photographs. **(A)** Case 1: initial left axillary mass with ulceration and surrounding edema. **(B)** Case 1: complete healing at 12-month follow-up. **(C)** Case 2: multiple subcutaneous nodules on forehead, BCG site, and left lower leg at presentation. **(D)** Case 2: resolution of nodules and healing of BCG scar at final visit.

On May 6, 2024, the infant underwent HSCT at approximately 4.5 months of age. One month post-HSCT, the left axillary mass enlarged, accompanied by bilateral upper limb swelling and new skin lesions. Based on multiple non-contiguous sites of involvement, mNGS detection of MTBC, no known *Mycobacterium tuberculosis* exposure, confirmed WAS, and recent BCG vaccination, the diagnosis was revised to clinically presumed disseminated BCG disease. Species-level identification was not available. The therapeutic regimen was adjusted to INH, EMB, and amikacin (AMK), supplemented with CZD (200 mg q12 h, oral, body weight 10 kg, subsequently adjusted to 12–20 mg/kg/dose). CZD was started 1 month post-HSCT, after the most severe aplastic phase. After 12 months of treatment, the axillary mass had completely healed (Figure 1B) with no significant abnormalities in blood counts or liver and kidney function tests. No CZD dose interruption or reduction was required. This represented clinical resolution. No clinical relapse was observed during 1-year post-treatment telephone follow-up. Serial microbiological clearance was not systematically documented.

### 2.2 Case 2

A male infant, aged 2 months 29 days, was admitted with a 5-day history of coughing and milk aspiration, along with a history of recurrent infections since birth. The patient received routine BCG vaccination at birth. Physical examination revealed erythema, rhagades, and desquamation on the hands and feet. Erythematous desquamative lesions were also observed on the occiput, neck, and trunk. Beaded follicles were present on the buccal and lower lip mucosa. Lymphocyte subset analysis indicated T-cell immunodeficiency, and whole-exome sequencing identified a hemizygous variant in the *IL2RG* gene (NM_000206.2: c.426del, p.Thr143Hisfs*4), confirming a diagnosis of X-linked severe combined immunodeficiency (X-SCID).

The patient underwent HSCT on November 2, 2023. Five days post-HSCT, he developed a fever. mNGS of the peripheral blood detected MTBC, also consistent with clinically presumed disseminated BCG disease in this post-vaccination patient with X-SCID. Species-level identification was not available. Consequently, anti-mycobacterial therapy consisting of INH, RFP, and LZD was initiated. One month into treatment, subcutaneous nodules were observed on the patient's left lower abdomen, forehead, left shin, and right elbow. Additionally, two masses appeared at the BCG vaccination site on the left upper arm (Figure 1C). Due to the potential drug-drug interaction between RFP and antifungal agents, and the risk of LZD-associated myelosuppression, the regimen was adjusted to INH, EMB, levofloxacin (LFX), and CZD (85 mg q12 h, oral, body weight 7 kg). Two weeks later, as the skin nodules showed a tendency to enlarge, the treatment was further intensified with moxifloxacin (MFX) and AMK, and increasing CZD dosage to 96 mg q12 h (subsequently adjusted to 12–20 mg/kg/dose). After 12 months of combination therapy, the subcutaneous nodules resolved, and the BCG scar healed (Figure 1D). During the treatment course, a mild transient elevation of blood urea nitrogen was observed. This event was not considered related to CZD and resolved without CZD discontinuation. No hematological toxicity or hepatic impairment was observed during the follow-up period. No clinical relapse was observed during 1-year post-treatment telephone follow-up. Serial microbiological clearance was not systematically assessed.

### 2.3 Case 3

A 9-month-15-day-old female was admitted due to a non-healing infection following the excision of a left axillary mass and recurrent fever. The patient had received routine BCG vaccination at birth. Genetic testing identified biallelic variants in the *IL12RB1* gene (NM_005535.2: c.1264C > T, p.Arg422Ter, inherited from the father; and c.517C > T, p.Arg173Ter, inherited from the mother), confirming a diagnosis of IL-12 receptor β1 deficiency (Mendelian susceptibility to mycobacterial disease, MSMD). MTBC was detected in gastric aspirate, blood, cerebrospinal fluid and axillary lymph node secretions. Notably, blood mNGS identified *Mycobacterium bovis* (BCG strain), which was resistant to pyrazinamide. This case was therefore classified as microbiologically confirmed *Mycobacterium bovis* BCG disseminated disease.

The patient initially received a multidrug anti-mycobacterial regimen (INH, RFP, LZD, and MFX), but due to persistent fever and severe anemia occurring during LZD therapy, with suspicion of LZD-associated hematological toxicity, LZD was replaced with CZD (120 mg q12 h, oral, body weight 7.5 kg, subsequently adjusted to 12–20 mg/kg/dose), and EMB was added. Hemoglobin was 71 g/L before LZD, decreased to a nadir of 64 g/L during LZD therapy, and remained above 80 g/L after switching to CZD. Because the patient also had severe systemic illness and received multiple concurrent medications, a definitive causal relationship between LZD and anemia cannot be established. The subsequent regimen consisted of INH, RFP, EMB, MFX and CZD. In January 2025, the patient underwent a 5/10 HLA-matched haploidentical HSCT from her father. However, primary graft failure occurred despite three infusions. Subsequent telephone follow-up revealed that the patient successfully underwent a second transplantation. The anti- mycobacterial therapy was discontinued after 11 months. No clearly attributable CZD-related adverse events were identified, although multiple concurrent medications and severe baseline illness complicated attribution. No clinical relapse was observed during 1-year post-treatment telephone follow-up. Serial microbiological clearance was not systematically assessed.

## 3 Diagnostic assessment, therapeutic intervention, and follow-up

All three patients underwent comprehensive immunological evaluation (Table 1) and genetic sequencing. mNGS provided rapid etiological diagnosis, which is particularly valuable in critically ill infants (9). Therapeutic interventions included tailored multi-drug antimycobacterial regimens and HSCT, the latter being the only curative modality for PID (10, 11). CZD was introduced either post-HSCT (Cases 1 and 2) or pre-HSCT (Case 3) due to LZD intolerance or suboptimal response.

**Table 1:** Clinical characteristics of three pediatric patients with clinically presumed or confirmed disseminated BCG disease treated with CZD-containing regimens.

Characteristics	Case 1	Case 2	Case 3
Sex/Age	Male, 3 months 20 days	Male, 2 months 29 days	Female, 9 months 15 days
BCG vaccination timing	At birth	At birth	At birth
Genetic diagnosis	*WAS* mutation	*IL2RG* mutation	*IL12RB1* mutations
Diagnostic classification	Clinically presumed	Clinically presumed	Microbiologically confirmed
Clinical presentation	Fever, ruptured axillary abscess, and new skin lesions post-HSCT	Pneumonia, post-transplant fever, respiratory distress, and skin/mucosal nodules	Ruptured abscess, recurrent fever, seizures, hepatosplenomegaly, pneumonia, meningitis, and HLH
Initial anti-mycobacterial regimen	INH + RFP + EMB	INH + RFP + LZD → INH + EMB + LFX	INH + RFP + LZD + MFX (for 2 weeks)
Rationale for CZD initiation	Suboptimal response to conventional therapy; disease progression post-HSCT	Prevention of LZD-induced myelosuppression; suboptimal response to conventional therapy	Persistent fever and severe anemia after initial therapy, with suspicion of LZD-associated hematological toxicity
CZD initiation time (relative to HSCT)	1 month post-HSCT (after aplastic phase)	−1 month post-HSCT	before HSCT (at −9 months of age)
CZD dosage and route	200 mg q12 h → adjusted to 12–20 mg/kg/dose; oral	85 mg q12 h → 96 mg q12 h (after 2 weeks) → adjusted to 12–20 mg/kg/dose; oral	120 mg q12 h → adjusted to 12–20 mg/kg/dose; oral
TDM performed	No	No	No
CZD-containing regimen	INH + EMB + AMK + CZD	INH + EMB + MFX + AMK + CZD	INH + RFP + EMB + MFX + CZD
CZD-related adverse events	None observed	None (mild transient BUN elevation, not attributed to CZD)	None clearly attributable (multiple concurrent medications)
CZD dose interruption/reduction	No	No	No
Follow-up duration	12 months treatment + 1 year post-treatment telephone follow-up	12 months treatment + 1 year post-treatment telephone follow-up	11 months treatment + 1 year post-treatment telephone follow-up
Outcome and prognosis	Clinical resolution; no clinical relapse during 1-year post-treatment telephone follow-up	Clinical resolution; no clinical relapse during 1-year post-treatment telephone follow-up	Successful second transplantation; Clinical resolution; no clinical relapse during 1-year post-treatment telephone follow-up

CZD, contezolid; INH, isoniazid; RFP, rifampicin; EMB, ethambutol; LZD, linezolid; LFX, levofloxacin; MFX, moxifloxacin; AMK, amikacin; HLH, hemophagocytic lymphohistiocytosis; TB, tuberculosis; HSCT, hematopoietic stem cell transplantation; TDM, therapeutic drug monitoring; BUN, blood urea nitrogen.

Disseminated BCG disease was defined as involvement of at least two non-contiguous sites or bloodstream/cerebrospinal fluid involvement in a patient with confirmed PID and recent BCG vaccination. Cases 1 and 2 were classified as clinically presumed disseminated BCG disease because species-level discrimination between *Mycobacterium tuberculosis* and *Mycobacterium bovis* BCG was not performed. Case 3 was classified as microbiologically confirmed *M. bovis* BCG disseminated disease based on species-level mNGS identification.

CZD was used off-label as part of individualized multidrug antimycobacterial regimens. The dose was extrapolated from adult weight-based regimens and institutional experience, targeting 12–20 mg/kg/dose every 12 h, without exceeding the adult dose. Dose adjustments were based on body weight, clinical response, and tolerability. Therapeutic drug monitoring was not performed.

In this retrospective series, treatment response was primarily assessed clinically. Clinical resolution was defined as defervescence, healing of skin and lymph node lesions, and weight gain. Serial microbiological monitoring by culture or mNGS from blood or other specimens was not performed after treatment initiation. Therefore, microbiological clearance and the time to pathogen negativity could not be determined. The observed outcomes should be interpreted as clinical resolution rather than microbiological cure.

During CZD therapy, blood counts, liver and renal function, electrolytes, lactate, and coagulation were checked at least weekly during hospitalization and every 2–4 weeks after discharge, with additional tests as indicated. At each visit, neurological assessment included developmental milestones and parental reports of seizures, abnormal tone, regression, visual and hearing changes, or peripheral neuropathy symptoms. Pediatric neurology consultation was obtained when indicated.

Follow-up durations were 12, 12, and 11 months from treatment initiation/CZD start to the last recorded follow-up. Antimycobacterial therapy was discontinued after 11–12 months. After treatment discontinuation, all patients were followed by telephone for an additional 1 year, and no clinical relapse was observed. Because serial microbiological monitoring was not performed, complete microbiological cure could not be confirmed, and relapse beyond 1 year cannot be fully excluded. A comprehensive clinical timeline is depicted in Figure 2, which integrates all key events from BCG vaccination to final follow-up.

**Figure 2:**
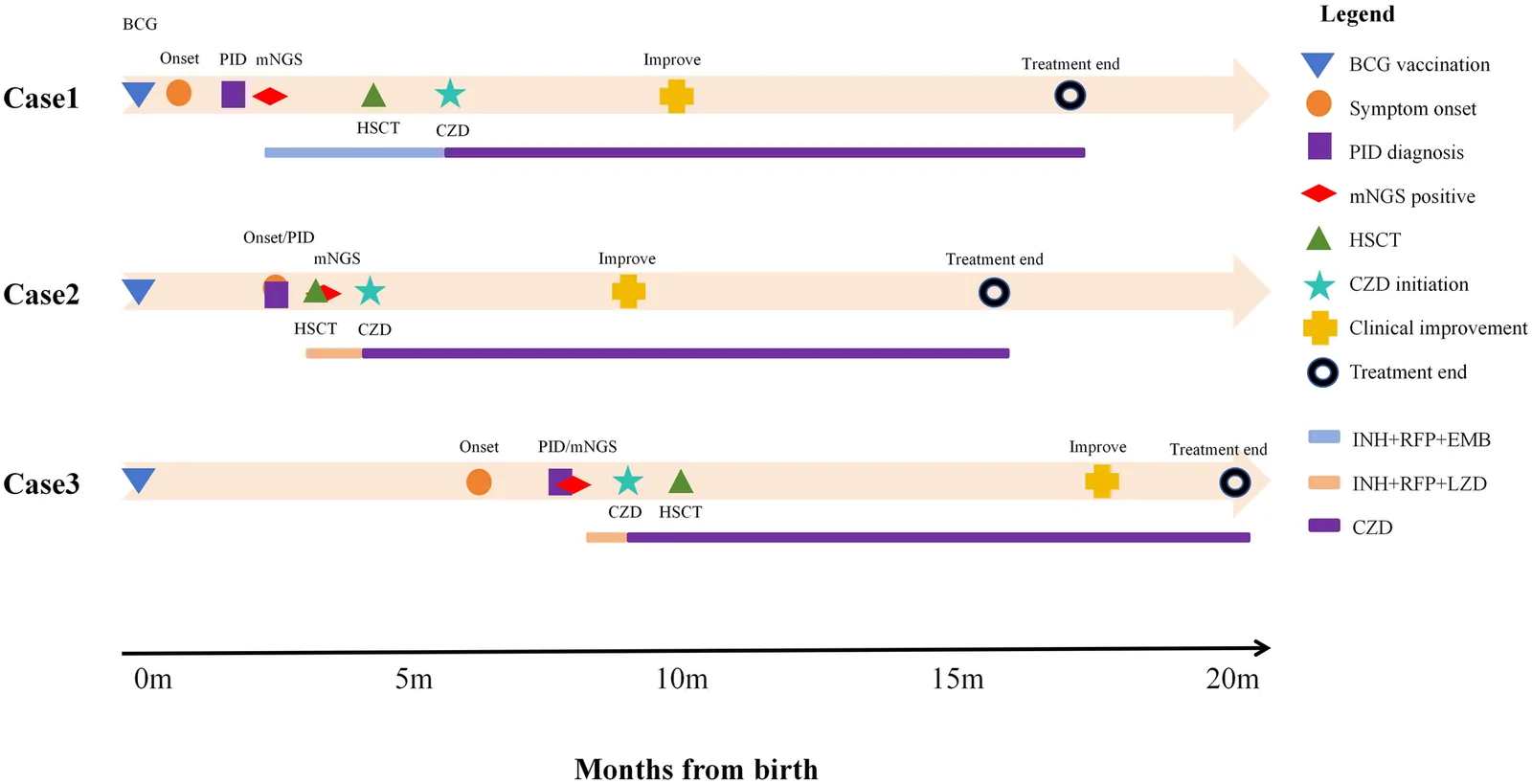
Patient-level clinical timelines for the three cases. Each horizontal bar represents the clinical course from birth to the end of follow-up. Key clinical events are marked along each timeline: BCG vaccination (▼), symptom onset (●), PID diagnosis (■), mNGS detection of MTBC (◆), hematopoietic stem cell transplantation (▲), CZD initiation (★), clinical improvement (┼), and treatment end (◎). The start and stop times of major antimycobacterial drugs, especially LZD and CZD, are also shown. The horizontal axis is scaled in months from birth. The timing of events was derived from medical records and reported by the treating clinicians. BCG, Bacillus Calmette–Guérin; PID, primary immunodeficiency; mNGS, metagenomic next-generation sequencing; MTBC, *Mycobacterium tuberculosis* complex; HSCT, hematopoietic stem cell transplantation; CZD, contezolid; LZD, linezolid.

## 4 Discussion

This retrospective case series provides detailed clinical experience on the use of CZD in three infants with clinically presumed or confirmed disseminated BCG disease and different PID genotypes. The most striking observation is the absence of significant hematological, neurological, or hepatic-renal toxicity attributable to CZD over a combined follow-up of 35 patient-months. This aligns with adult data showing lower rates of anemia and neuropathy compared with LZD (3, 12, 13). In our cases, LZD had to be replaced in Case 3 due to severe anemia, and in Case 2 it was changed prophylactically to avoid myelosuppression in the post-HSCT setting. The favorable tolerability of CZD in these vulnerable children suggests it may be a safer oxazolidinone alternative when prolonged therapy is required.

All three patients underwent HSCT, which is currently regarded as the definitive curative modality for PIDs capable of reconstituting immune function (10). Patients with PIDs are highly susceptible to disseminated, life-threatening BCG and non-tuberculous mycobacterial infections. Several PID subtypes, including SCID, CGD, and MSMD, can be cured through HSCT (11). The immune reconstitution effect of HSCT therefore constitutes a significant confounding factor, precluding unequivocal attribution of the observed clinical improvements to the direct anti-mycobacterial activity of the CZD-containing regimen. In Cases 1 and 2, new or worsening lesions appeared after HSCT, suggesting possible BCG-related immune reconstitution inflammatory syndrome (BCG-IRIS) (14). In Case 1, the axillary mass enlarged 1 month post-HSCT; in Case 2, subcutaneous nodules appeared at a similar time. Both patients had elevated inflammatory markers and persistent positive mNGS results at progression. During this period, the anti-mycobacterial regimen was modified to include CZD and no additional immunosuppressive agents were introduced, after which the lesions gradually improved.

Post-HSCT lesion progression may represent BCG-IRIS rather than microbiological treatment failure. BCG-IRIS is typically characterized by paradoxical clinical worsening with new or enlarging lesions following immune reconstitution, often accompanied by elevated inflammatory markers, in the absence of an alternative diagnosis (14, 15). In contrast, true microbiological progression may show persistent positive cultures or mNGS and continued clinical deterioration. Distinguishing BCG-IRIS from true infection progression is challenging and often requires a diagnosis of exclusion. Inflammatory biomarkers and tissue pathology may be helpful (15, 16). In our cases, mNGS remained positive at progression, and no additional immunosuppression was given. Therefore, we could not definitively distinguish BCG-IRIS from true microbiological progression. The subsequent improvement after regimen modification including CZD may also reflect immune reconstitution and other antimycobacterial agents. This uncertainty further limits attribution of benefit to CZD alone.

The potential advantage of CZD over LZD in reducing myelosuppression during the post-HSCT aplastic phase remains inadequately substantiated. None of the three cases received CZD during the early post-HSCT period when marrow aplasia was at its nadir. In Cases 1 and 2, CZD was initiated only after the most severe cytopenic phase had passed, while in Case 3 it was started before transplantation. Thus no direct evidence is provided that CZD is safer than LZD during this high-risk setting. To date, only one post-HSCT case of pulmonary tuberculosis has been reported, in which CZD was initiated after the most severe myelosuppression had already resolved (17). No prospective study has evaluated CZD during the pre-engraftment aplastic window. Thus, whether CZD confers measurable clinical benefit during the highest-risk aplastic phase remains unknown, representing a key knowledge gap.

We acknowledge several important limitations. The retrospective nature of this study, together with the small cohort size and the relatively limited observation window, precludes any firm conclusion regarding CZD's efficacy in this population. Moreover, because all three patients received concomitant HSCT and multi-agent regimens, the independent anti-mycobacterial contribution of CZD cannot be reliably gauged. Diagnostic precision was also incomplete. While mNGS confirmed the presence of MTBC in Cases 1 and 2, species-level discrimination between *Mycobacterium tuberculosis* and *Mycobacterium bovis* BCG was not performed, and conventional culture with drug susceptibility data was unavailable throughout. Cases 1 and 2 were therefore classified as clinically presumed disseminated BCG disease. Another limitation concerns outcome ascertainment. Treatment response was assessed clinically, and serial microbiological monitoring (culture or mNGS) was not performed after treatment initiation. Therefore, microbiological clearance and time to pathogen negativity could not be determined, and outcomes should be interpreted as clinical resolution rather than microbiological cure. After treatment discontinuation, 1-year telephone follow-up showed no clinical relapse, but microbiological cure could not be confirmed and late relapse beyond 1 year cannot be excluded. In addition, CZD serum concentrations were not measured, so the adequacy of the pediatric dosing schedule remains untested. Larger, prospectively designed studies with standardized pharmacokinetic monitoring and longer observation are needed to define the optimal pediatric CZD regimen, to confirm its long-term safety, and to identify the most rational companion agents.

## 5 Patient perspective

The parents or guardians of all three children expressed satisfaction with the treatment outcomes. They observed that the visible skin lesions, fever, and general distress of their children gradually diminished, which provided reassurance during a prolonged and stressful course. They appreciated the clear communication from the medical team and willingly consented to publication, hoping to help other families facing similar situations.

## Data Availability

The datasets presented in this article are not readily available because of ethical and privacy restrictions. Requests to access the datasets should be directed to the corresponding authors.
